# Supplementary material for: Guiding visual attention in deep convolutional neural networks based on human eye movements
Source: Front Neurosci. 2022 Sep 13;16:975639. doi: 10.3389/fnins.2022.975639 (PMC9514055; doi:10.3389/fnins.2022.975639)
Supplement: Supplementary file 1 [file Data_Sheet_1.pdf]

Supplementary Figures

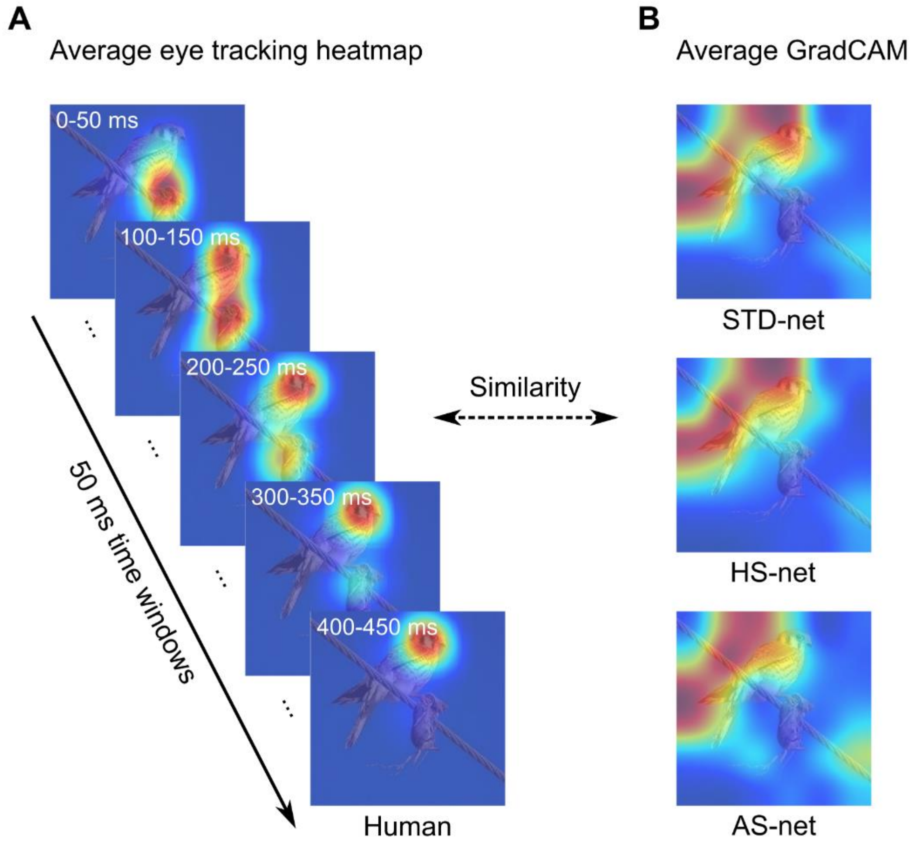

Supplementary Figure 1

Image example with average visual attention of human participants and DCNN manipulation types. **(A)** Average human eye tracking heatmaps across 50 ms time windows. **(B)** Average DCNN GradCAM saliency maps for individual manipulation types.

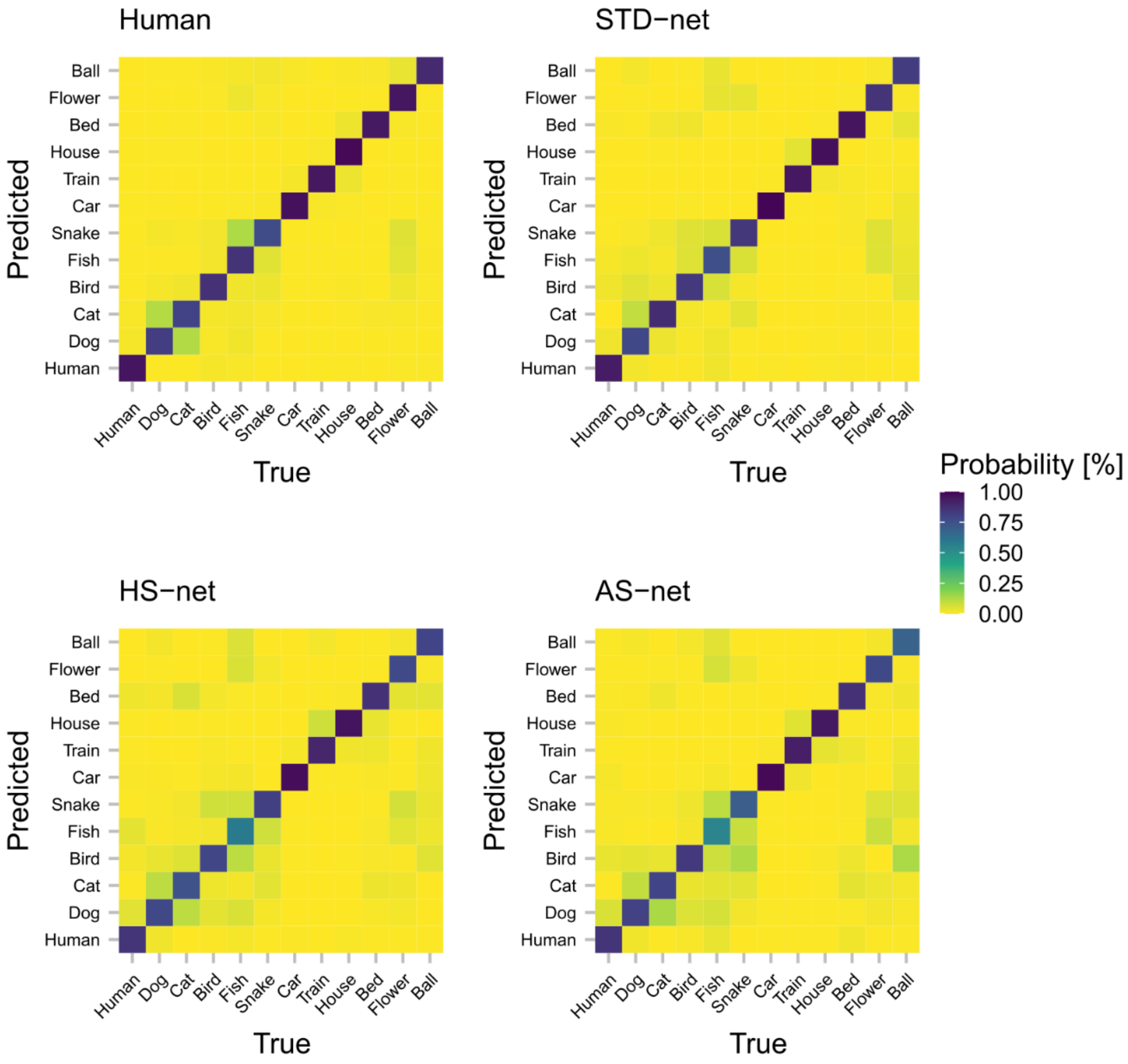

Supplementary Figure 2

Confusion matrices displaying common miscategorizations by probability of occurrence for true versus predicted category combinations in human participants, standardly fine-tuned STD-nets, purposefully manipulated HS-nets, and inversely manipulated AS-nets across all testing images.

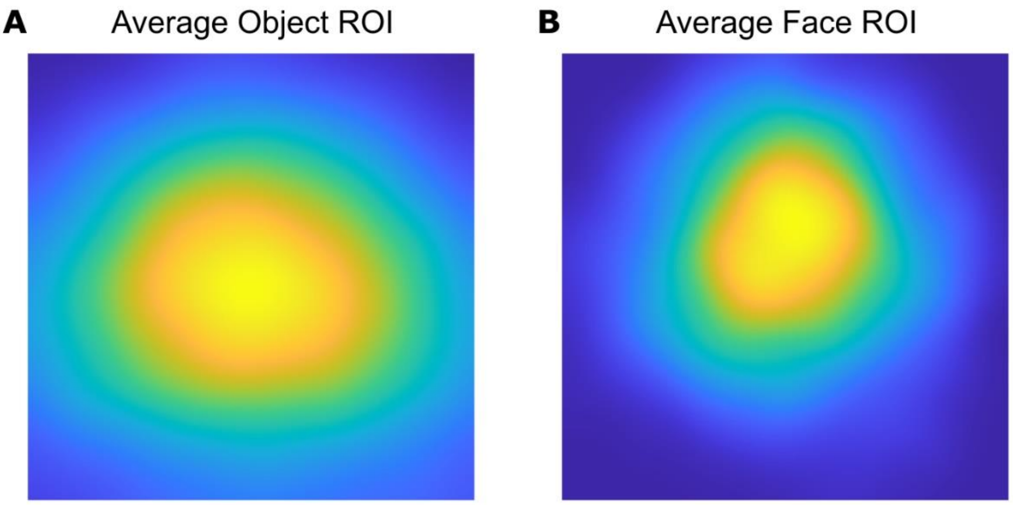

Supplementary Figure 3

Average manually segmented regions of interest (ROIs). **(A)** Average object ROI (N = 360). **(B)** Average face ROI (N = 133).
